# Pediatric ACL Reconstruction in Children—An Evaluation of the Transphyseal Technique’s Efficacy and Safety

**DOI:** 10.3390/children11050545

**Published:** 2024-05-03

**Authors:** Alexandru Herdea, Mihai-Codrut Dragomirescu, Valentin Burcan, Alexandru Ulici

**Affiliations:** 1 11th Department of Pediatric Orthopedics, “Carol Davila” University of Medicine and Pharmacy, Bd. Eroii Sanitari Nr. 8, 050474 Bucharest, Romania; alexandru.ulici@umfcd.ro; 2 Pediatric Orthopedics Department, “Grigore Alexandrescu” Children’s Emergency Hospital, 011743 Bucharest, Romania; mcodrutdragomirescu@spitalulgrigorealexandrescu.ro (M.-C.D.); valentin.burcan@stud.umfcd.ro (V.B.)

**Keywords:** ACLR, pediatric, adolescent, outcomes, knee instability, skeletally immature

## Abstract

Introduction: Injuries of the anterior cruciate ligament (ACL) are commonly found in the general population, both among adult and pediatric patients, and their incidence has been increasing in recent years. Most of the late literature agrees that surgical reconstruction of the ACL is effective in improving long-term outcomes in pediatric patients, while others in the past have pleaded for non-surgical management. Purpose/Hypothesis: Our study aims to verify if ACL reconstruction (ACLR) using transphyseal technique in skeletally immature patients will provide angular deviations or growth restrictions. Study Design: Retrospective cohort study; Level of evidence 4. Methods: We perfomed a retrospective study to verify if transphyseal ACLR in children with less than or equal to 2 years of remaining growth leads to either limb length discrepancies or axis deviations. Results: Most patients who were treated using transphyseal technique showed significant improvements in their functional scores. There were statistically significant differences in lateral distal femoral angles (LDFA) and medial proximal tibial angles (MPTA), with no clinical impact. There was no significant limb length discrepancy (LLD) during the 2-year follow-up. Conclusions: Transphyseal ACLR is safe among children who have less than or equal to 2 years of remaining growth and brings no risk of axis deviations or limb length discrepancy.

## 1. Introduction

Anterior Cruciate Ligament (ACL) tears are a significant concern, especially among athletes, and often occur in conjunction with other knee injuries, such as meniscal lesions and damage to other ligaments. Early and accurate diagnosis is crucial, as well as effective early treatment [1,2,3,4]. The incidence of ACL reconstructions (ACLR) has increased recently, indicating a rise in ACL injuries. This increase is particularly notable among younger athletes, highlighting the need for effective prevention and treatment strategies [5]. ACL injuries can lead to long-term issues such as osteoarthritis, even after surgical reconstruction [6,7]. The low rate of getting back into sports after ACLR is a significant concern, with evidence suggesting that delaying return to sports until approximately 2 years post-surgery may lower the risk of subsequent ACL injuries, especially among younger, more active athletes [8].

There are both surgical and non-operative treatment options for ACL tears in children, and each is customized to the patient’s specific needs depending on factors like bone maturity and activity level. Anatomic reconstruction is possible with transphyseal ACLR. The youngest children with the greatest amount of growth remaining should consider physeal-sparing ACL repairs because they avoid the need for bone tunnels that cross the physis [9]. As early as 1986, partial transphyseal ACLR were reported using a transphyseal tibial tunnel and over-the-top physeal-sparing femoral graft fixation [9]. The advantage of all-epiphyseal ACL restorations is that they restore the anatomic footprint of the ACL while providing benefits akin to those of over-the-top iliotibial band reconstruction [9].

Long-term follow-up of children who have undergone ACL surgery is mandatory to monitor for growth disturbances and other complications. Our study aims to verify if transphyseal ACLR in skeletally immature patients will provide angular deviations or growth restrictions. We hypothesized that transphyseal ACLR among children with less than or equal to 2 years of remaining growth is a safe procedure with no risk of significant malalignment or limb length discrepancy.

## 2. Materials and Methods

### 2.1. Study Design

**Figure 1:**
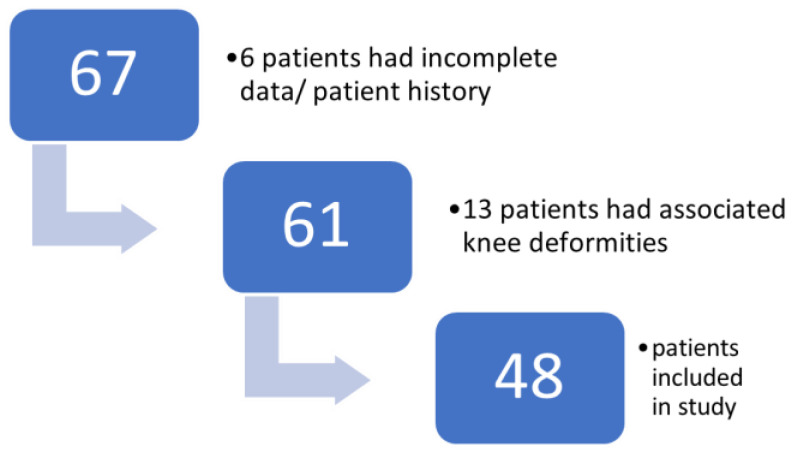
Flow diagram that shows the patient selection prior to data analysis.

The investigation was conducted at the Pediatric Orthopedics Outpatient Clinic situated within the “Grigore Alexandrescu” Children’s Emergency Clinical Hospital in Bucharest, Romania. This clinic serves as a primary healthcare facility for children in the urban vicinity and its surroundings. Approval for the study was obtained from the hospital ethics committee on 26 September 2023, and the survey was assigned the identification number 14. Written consent was secured from each parent or legal tutor of all participating children. The study covered the period from January 2018 to January 2022 through consecutive sampling. Figure 1 provides a patient flow diagram that outlines the study design and the process of participant selection.

### 2.2. Participants

A retrospective single-center investigation took place from January 2018 to January 2022. The study involved children ranging from 12 to 16 years old who were referred to the clinic due to knee trauma with ACL injury and had subsequently undergone ACLR.

The inclusion criteria comprised a positive diagnosis of ACL injury confirmed through knee MRI, a clinical examination supporting the diagnosis, less than 2 years of remaining skeletal growth (conventionally determined by radiographical assessment using middle phalanx of third finger, Risser and Greulich and Pyle methods), and a follow-up of 2 years or more.

The exclusion criteria encompassed the absence of patient history, concomitant knee deformities (as genu varum, genu recurvatum, deformities that might interfere with our measurements) or local comorbidities, absence of informed consent, high-energy trauma involving the physes, incomplete data, follow-up duration of less than 2 years, and the presence of local comorbidities.

### 2.3. Study Procedure

Following anamnesis and a thorough clinical examination related to knee trauma, X-ray and MRI assessments were conducted. Initial lateral knee X-rays and orthoroengenograms were performed. Subsequently, patients were scheduled for knee MRI. During the period between the initial assessment and the receipt of MRI results, full weight-bearing was restricted. Patients were instructed to engage in toe-touch weight bearing while using a knee orthosis, maintaining 0-0 flexion-extension.

The preoperative evaluation of the patients involved a comprehensive history and physical examination, including the Tegner activity score, Tegner-Lysholm score, orthoroentgenograms, knee MRI scans, and laboratory tests.

**Figure 2:**
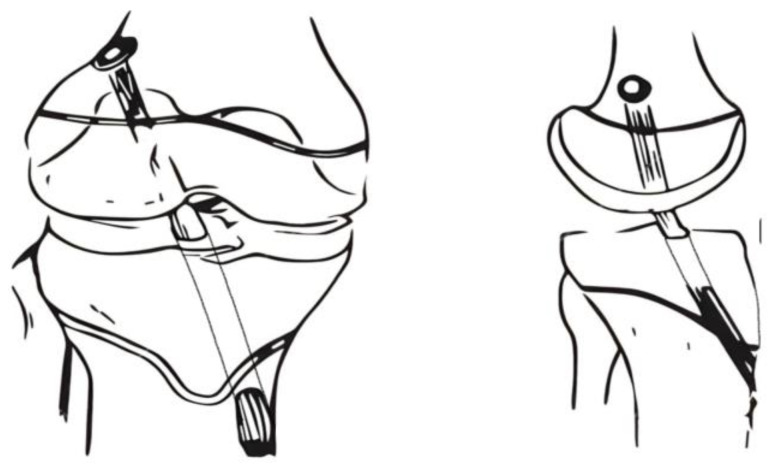
Illustration of the femoral and tibial tunnels in coronal (**left**) and sagittal (**right**) planes.

Transphyseal ACLR was performed using the same technique for every patient, by the same surgical team, after the confirmation of total ACL rupture through MRI results. The tibial tunnel was performed in an outside-in manner, and the femoral tunnel using the anteromedial portal technique. Figure 2 illustrates the placement and orientation of femoral and tibial tunnels.

ACLR was performed with hamstring tendons used for graft preparation. The femoral side was secured with a button, and the tibial side was stabilized with an absorbable interference screw. Any coexisting meniscal injuries were addressed during the same surgical procedure with all-inside or outside-in suture techniques, neither reaching the growth plate. All patients followed a consistent postoperative protocol, including the use of an adjustable knee orthosis, partial weight bearing, and physical therapy. Follow-up assessments were carried out at 2 and 4 weeks, 3, 6, 12, and 24 months.

Follow-up was conducted for at least two years for each patient, comprising clinical examination, X-rays, Tegner-Lysholm score, and Tegner activity score, all used to measure the outcome. Standing AP view of lower limbs was assessed every 6 months in the remaining growth time. Functional scores were assessed 6 months postop, and 2 years later.

### 2.4. Statistical Analysis

Information was gathered and stored in the institutional informatics system. IBM^®^ SPSS^®^ Statistics (Version 26) and Microsoft Excel Office 2016 (Microsoft, Redmond, Washington, DC, USA) were utilized for data analysis. The data encompassed categorical qualitative variables such as the type of lesion, sex, Tegner-Lysholm score, and Tegner’s activity score, as well as continuous quantitative data, including age at evaluation, mechanical axis deviation (MAD), anatomic lateral distal femoral angle (aLDFA), medial proximal tibial angle (MPTA), and limb length discrepancy (LLD) at 2 year follow-up.

Kolmogorov-Smirnov test was used to test normal distribution and the results (KS Statistic, *p*-value) indicated that the data was normally distributed for each variable. Chi-square test and paired T test were conducted, with 95% confidence intervals. Statistical significance was attributed to results where the *p*-value was less than 0.05.

## 3. Results

A database was conducted with the cases of 48 skeletally immature pediatric patients who received orthopedic surgery utilizing the conventional transphyseal technique between 2018 and 2022. The sample consisted of 27 male (56.25%) and 21 female patients (43.75%), ranging in age from 12 to 16 years old. The mean age at diagnosis was 14.08 years. For the sample of boys, the mean age was 14.42 years (S.D. ± 0.98), while the mean age for girls was 13.8 years (S.D. ± 0.175). Apart from chronological age, skeletal age was conventionally measured on X-rays using middle phalanx of third finger (if fused, it means there is less than 1 year of growth remaining), Risser (Risser 1 occurs after menarche in girls and precedes growth maturity with 2 years), and Greulich and Pyle methods. All cases were identified as having ACL injuries resulting from accidental trauma during athletic competitions, skiing, or physical education classes.

The average time between the lesion and the positive diagnosis was 14.125 weeks, while between the diagnosis and the surgery the average time was 4.02 weeks. At the time of the study, only 8 out of 48 (16.67%) patients resumed participating in sports within a mean of 30 weeks.

**Figure 3:**
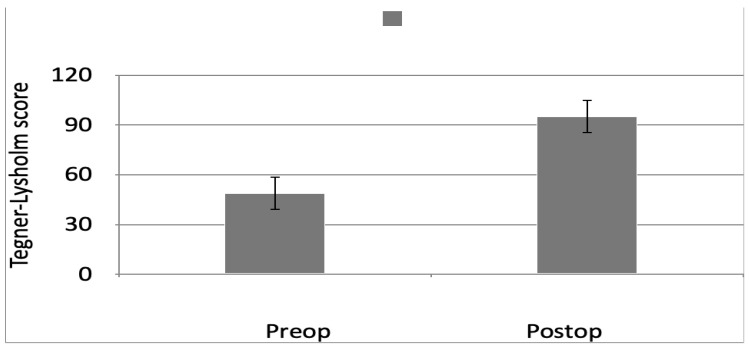
Comparison of preoperative and postoperative Tegner-Lysholm score.

The preoperative and postoperative clinical evaluation was conducted using the Tegner-Lysholm score, with the average preoperative score being 71.9, which improved to 93.08 after 12 months of arthroscopic ACLR, highlighting that nearly all the patients have shown improvement after the surgery with no instability during routine and exertional activities. Figure 3 illustrates the comparison of Tegner-Lysholm scores. We also compared preoperative and postoperative Tegner’s activity scores, which showed a statistically significant reduction, as shown in Table 1. No re-ruptures were observed during follow-up. The quality of life of the patients became similar to the one before the trauma. None of the patients manifested instability throughout the postoperative care, according to the Lachman, drawer, and pivot-shift tests.

As illustrated in Table 2, there were differences between operated and contralateral LDFA and MPTA angles that were statistically significant (*p* < 0.001), as well as for MAD and LLD.

Regarding MAD, there was a mean difference of 1.3750 mm, with a minimum of 1 mm and a maximum of 5 mm, having a standard deviation of 1.4086 and a 95% percentile of 3.55.

The mean difference of aLDFA was 0.68750, with a minimum of 0 mm and a maximum of 3 mm. SD was 0.74822. 95% percentile calculated was 2 mm. Regarding MPTA, the average was 0.70833 mm, between a minimum of 0 and a maximum of 3 mm, with a SD of 0.79783 and a 95% percentile of 2.55 mm.

LLD difference at 24 months postoperatively measured on the orthoroentgenogram was 1.3958 mm on average, ranging from 0 to 8 mm, with a statistically significant variation (*p* < 0.001).

## 4. Discussion

Our study’s findings reveal that anterior cruciate ligament (ACL) reconstruction in pediatric patients yields favorable outcomes, as evidenced by significant improvements in both the Tegner-Lysholm score, which assesses knee function and symptoms, and the Tegner activity level score, which measures activity capacity. Also, as our aim has been met, the surgical intervention demonstrated a notable absence of clinically relevant deformities post-procedure.

ACL lesion among children is still a source of debate regarding operative or conservative management and most of the authors emphasize the deficiencies in the literature. In the early literature, it was believed that conservative management is adequate in skeletally immature patients, followed by reconstruction after physeal closure [9]. There is evidence that patients who postpone surgery face the risk of additional lesions, such as cartilage and meniscus tears. Even nowadays, for children younger than 12, there are authors that suggest non-operative treatment as the best option [10]. For patients who are physically active, research indicates that conservative treatment can lead to secondary injuries to the meniscus and cartilage, as well as accelerated degenerative changes. This outcome persists despite the inclusion of rehabilitation exercises and the use of orthotic bracing within the treatment regimen [11]. Surgical techniques have been refined to reconstruct the anterior cruciate ligament (ACL) while minimizing the potential risks to the growth plate (physis) [12]. In our practice, ACL lesions of 50% or more are immediately prone to reconstruction, while partial lesions under 50% of the transverse diameter undergo conservative management and rehabilitation.

Operative treatment, rather than nonoperative treatment, is considered the standard of care for even the youngest athletes with an ACL injury [13]. Physeal sparing (extraphyseal and all-epiphyseal), partial transphyseal, and transphyseal reconstructions are all acceptable techniques based on the patient’s skeletal age, physician preference, and shared decision-making [14].

Operative intervention, as opposed to nonoperative management, is recognized as the standard of care for even the youngest athletes experiencing an ACL injury [13]. Techniques such as physeal sparing (including extraphyseal and all-epiphyseal approaches), partial transphyseal, and complete transphyseal reconstructions are deemed appropriate based on the patient’s skeletal maturity, the surgeon’s preference, and the shared decision-making [14].

A logical strategy for managing this issue involves assessing the relative risk (high, intermediate, or low) by evaluating the patient’s chronological age, skeletal age, and physiological age [15,16]. The existing body of literature indicates that both early and delayed ACLRs are effective in restoring knee stability. However, postponing the reconstruction heightens the likelihood of meniscal injuries, with an increased risk of these tears being irreparable [17]. Nonoperative management of anterior cruciate ligament (ACL) injuries has been associated with a significant prevalence of secondary meniscal pathology, ongoing knee instability, and diminished rates of returning to athletic activities [17]. Therefore, it appears that performing early ACLR shows better results in IKDC and Tegner-Lysholm scores.

Kannus and colleagues found in their 8-year follow-up of conservative management for adolescents with ACL injuries that complete, unrepaired ACL lesions (grade III) had poor results and signs of osteoarthritis, and thus were not a viable option [18]. They concluded that surgical intervention should be administered to every patient with remaining growth who exhibits a complete lesion [18]. The study also found that grade I and II injuries were suitable for non-operative treatment and had acceptable results [18]. Compared to their results, we consider every patient with a complete lesion a candidate for ACLR and also every patient with ACL lesion of more than 50% of the diameter.

Mohtadi et al., in their systematic review, determined that there was insufficient evidence from randomized controlled trials to conclusively decide whether surgical or conservative management is the superior approach for treating ACL injuries [19]. The general agreement was that it was unlikely that younger patients would be able to effectively use bracing and maintain a limited level of activity [19]. Their management algorithm was based on skeletal maturity, concomitant meniscal damage, and the ability to follow a non-surgical treatment plan until skeletal maturity was reached [19].

In concordance to our results, McConkey and colleagues stated that reports of post-op growth disturbances in immature patients are uncommon and usually caused by surgical mistakes, such as placing hardware or bone plugs across the growth plate [20]. Clinical and animal studies suggest that the risk of growth disturbances can be reduced by making small transphyseal tunnels and utilizing a soft tissue graft to fill them, avoiding the use of fixation devices and bone plugs near the growth plate, and preventing over-tensioning of the graft [20]. We found in our patient pool minimal axis deviations and discrete LLD without clinical significance.

As reported by Perkins and colleagues, for males with a bone age of 13 to 14 years and females with a bone age of 11 to 12 years, a transphyseal reconstruction can be a suitable option. However, for boys under 12 and girls under 10, physeal-sparing techniques are generally recommended [21]. We used a similar landmark: transphyseal reconstruction for patients having 2 years of less of growth remaining.

According to a study by Vavken et al., over fifty percent of children and adolescents who underwent surgery for an ACL tear had additional intra-articular knee injuries that required further treatment. These injuries, which may include meniscal tears, cartilage damage, or other ligament injuries, can affect the overall recovery process [22]. In addition, research by James et al. in 2021 found that delaying ACL surgery 3 months or more in pediatric and adolescent athletes substantially elevated the risk of meniscal injuries and irreparable meniscal tears, yet both early and delayed surgical interventions produced favorable outcomes. Conversely, conservative treatment was associated with increased instances of knee instability and reduced rates of returning to sports activities [23].

Kiani et al. noted that the number of patients undergoing ACLR during the pandemic was lower than anticipated based on pre-pandemic trends. This reduction was likely attributed to a decrease in injury rates combined with delays in surgical procedures. It is important to conduct further research to evaluate the impact on these patients [24].

Wong et al., in a 2017 meta-analysis, concluded that growth disturbances may occur with any ACLR technique or graft selection, and that the precision of the surgical method might be more critical than the choice of reconstruction technique itself [25]. In our study, every patient was operated on by the same surgical team, and with the same technique, leaving less room for bias regarding the surgical method. The majority of ACL injuries in skeletally immature athletes tend to occur in adolescents who have minimal remaining growth potential (less than one year), which permits the use of conventional transphyseal reconstructions with a minimal risk of growth disturbances [21,26].

According to a meta-analysis by Kay and colleagues, children and adolescent athletes have a high rate of returning to sports following ACLR [27]. Nevertheless, this is associated with a relatively high incidence of graft rupture and a comparable rate of injury to the contralateral side [27]. Astur et al. observed that during mid-term follow-up, patients who experienced a re-rupture of the ACL had lower Tegner and Lysholm scores at both 6 and 9 months compared to those without a re-rupture [28]. They further noted that 77.8% of ACL re-ruptures occurred in patients younger than 20 years old, and 66.6% of these re-ruptures took place over 24 months after the initial ACLR [28]. In our period of two years postoperatively, there were zero incidences of graft rupture, but the lack of long-term follow-up after skeletal maturity limits our perspective on re-rupture rate and it’s correlation with clinical scores, thus leaving room for further research.

Recent studies about platelet-rich plasma (PRP) as an adjuvant in ACLR and in knee trauma concluded that when utilized in conjunction with ACLR, PRP may alleviate postoperative pain and enhance knee functionality over short- to medium-term periods with no extent into the long-term outcome, nor the knee stability [29,30]. During our follow-up of patients treated solely with ACLR, there were no noticeable instances of knee pain or restricted movement (regarding the post-op protocol of rehabilitation) that could be considered clinically significant. The fact that this lack of adverse outcomes occurred after anterior cruciate reconstruction using a transphyseal technique makes this method of reconstruction a more desirable option for older pediatric patients with an ACL tear.

One potential limitation of our study is that a significant proportion of the patients (40%) were not athletes, which may limit the ability to follow up with these patients and assess their long-term outcomes in exertional lifestyles. This could impact the generalizability of our findings, as the experiences and outcomes of non-athletic patients may differ from those of athletes. Given that only 8 out of 48 (16.67%) patients resumed sports after the surgery, it appears that kinesiophobia affects the majority of patients, even after restitutio ad integrum. This limitation should be considered when interpreting the results of our study, and it may be worth exploring ways to collect additional follow-up data on non-athletic patients in future studies. Another limitation is the lack of objective means to test the ligamentous stability of the knee, such as KT1000, a device that allows for accurate measurement of anteroposterior tibial translation during drawer test. Future studies could use similar apparel to quantify the quality of the surgery both during and after the intervention.

## 5. Conclusions

Both early and delayed ACLR demonstrate benefits for children based on functional scores and quality of life measurements.

The safety of transphyseal ACLR is affirmed in children with two years or less of remaining growth.

Transphyseal ACLR brings no significant risk of limb length discrepancy or axial deviation.

## Figures and Tables

**Table 1:** Preoperative and postoperative values of the Tegner-Lysholm score and Tegner’s activity score. Abbreviations: CI, confidence interval; ES, effect size; IKDC, International Knee Documentation Committee; LB, lower bound; SD, standard deviation; UB, upper bound. Note: t: result of paired sample *t*-test; p: expressed as a 95% CI. *p* < 0.001, ES: Cohen’s d effect size.

	Preoperative Mean ± SD	Postoperative Mean ± SD	*p*-Value	ES	95% CI	
					LB	UB
Tegner-Lysholm score	71.9 ± 5.47	93.08 ± 3.12	0.000 ^a^	4.75	19.37	22.98
Tegner’s activity score	6 ± 1.02	5 ± 1.06	0.000 ^a^	0.96	0.58	1.42

^a^ *p* < 0.001.

**Table 2:** Illustrating aLDFA, MPTA, MAD differences and LLD respectively between operated knee and contralateral. Legend: Δ = difference between operated and contralateral knee.

	ΔMAD	ΔaLDFA	ΔMPTA	LLD
Mean	1.3750	0.68750	0.70833	1.3958
Median	2	1	1	0.5
Minimum	1	0	0	0
Maximum	5	3	3	8
SD	1.4086	0.74822	0.79783	1.7593
95% Percentile	3.55	2	2.55	5

## Data Availability

The raw data supporting the conclusions of this article will be made available by the authors on request.
